# The Proposed Registration of Nurses

**Published:** 1889-12-07

**Authors:** 


### The Proposed Registration of Nurses.

The authorities responsible for the training and supply of nurses throughout the country have been astonished at the persistence with which it has been recently asserted that they, or the majority of them, are in favour of the scheme of voluntary registration for nurses advocated by the British Nurses' Association. In these circumstances we have been requested to publish the following protest of the largest and most important institutions throughout the United Kingdom against the scheme in question. We are informed that the unanimous feeling of the most practical and experienced persons, who have made nursing what it is to-day, is, that any system of voluntary registration, such as that proposed, must prove harmful rather than advantageous to both the public and the nurses, especially as the latter are to be asked to contribute to the expenses of the proposed registration out of their already too limited and relatively small earnings. Considerations of space prevent us from publishing more than a selection from the list of signatories already attached to this weighty protest, but they constitute not only the most representative list of hospital and nursing managers and teachers ever published, but afford overwhelming evidence that the practical knowledge and experience of those best capable of giving a sound opinion are entirely opposed to the voluntary register for nurses advocated by the British Nurses' Association. It is desirable that this fact should be plainly stated and widely circulated throughout the country. Such an end can, it is urged, be best attained by opposing sober facts to the poetic fictions so often promulgated by the enthusiastic partisans of a dying cause.

### MEMORIAL OF NURSE-TRAINING SCHOOL AUTHORITIES.

We, the undersigned, beg the favour of your insertion of the following statement, which we think it desirable to make, in view of a paragraph which has been published on the subject of the registration of nurses, in which we note with surprise the statement that the main object of the British Nurses' Association "is in conformity with a great public want, and a widespread professional demand."

We would wish to point out that those who represent the largest nursing interests in the metropolis and throughout the country, and who have the most to do with the training and examination of nurses, have not only declined to take part in the association, but consider that its proposed enrolment of nurses in a common register, if carried out, would (1) lower the position of the best trained nurses, (2) be detrimental to the advancement of the teaching of nursing, (3) be disadvantageous to the public, and (4) be injurious to the medical practitioner.

We hope that a final judgment upon this important matter will be postponed until the views of those who are opposed to the aims of this association have been expressed and examined. We further consider it our duty to state that if a charter be applied for on the lines stated in the prospectus of the British Nurses' Association, we shall feel it to be incumbent upon us to offer thereto all legitimate opposition in our power.

### First List London Hospital Authorities.

**St. Thomas's Hospital and Nightingale Fund Training School.**

D. H. Stone, treasurer of the hospital.
Harry Verney, chairman of the Nightingale School.
W. Bowman, F.R.S., member of council of Nightingale Fund, and of council of St. John's House and Sisterhood.
W. Rathbone, trustee and member of Council Nightingale Fund; president Liverpool Training School and Home for Nurses.
H. Bonham-Carter, secretary of the Nightingale Fund.
J. S. Bristowe, M.D., F.R.S., senior physician of St. Thomas's Hospital, and lecturer in Nightingale School.
A. L. Pringle, matron of St. Thomas's Hospital, and superintendent of Nightingale Fund Training School.
M. S. Crossland, sister in charge of the Nightingale Training School.

**Guy's Hospital and Training School.**

E. H. Lushington, treasurer.
E. C. Perry, M.D.; G. Newton Pitt, M.D.; assistant physicians and instructors of probationer nurses.
J. C. Steele, M.D., superintendent and instructor of nurses.

**Westminster Hospital and Training School.**

Westminster, chairman.
Rutherford Alcock, vice-chairman.
J. J. Troutbeck, D.D., hon. treasurer.
Mary E. Thynne, hon. secretary of Committee of Management of Training School.
W. H. Allchin, M.B., physician to the hospital; Thomas Bond, F.R.C.S., surgeon to the hospital; lecurers to the nursing staff.
Mary J. Pyne, matron of hospital and lady superintendent of nurses.

**St. Bartholomew's Hospital and Training School.**

Norman Moore, M.D., assistant physician; Harrison Cripps, F.R.C.S., assistant surgeon; instructors of probationary nurses, St. Bartholomew's Hospital.

**Charing Cross Hospital and Training School.**

John B. Martin, treasurer and chairman of committee.
Frederick Willcocks, M.D., assistant physician and lecturer to nurses.
Stanley Boyd, F.R.C.S., senior assistant surgeon and lecturer to nursing staff.
Hughina A. C. Gordon, lady superintendent.

**King's College Hospital and Training School.**

Henry Wace, D.D., chairman of Committee of Management.
Richard Twining, treasurer.
Nathaniel Bromley, A.K.C., secretary.
John Curnow, M.D.; Nestor Tirard, M.D.; physicans to the hospital, and examiners and lecturers to the nursing staff.
Katherine H. Monk, matron.
Clara S. A. Peddie, house sister and teacher to the nursing staff.

**London Hospital and Training School.**

F. C. Carr-Gomm, chairman of House Committee.
J. H. Buxton, treasurer.
A. Ernest Sansom, M.D.; Frederick Treves, F.R.C.S.; James Anderson, M.D.; examiners and lecturers to the nursing staff.
Eva C. E. Lückes, matron.

**St. Mary's Hospital and Training School.**

T. Pycroft, chairman of House and Finance Committee.
M. Handfield Jones, M.D., assistant obstetric physician; A. J. Pepper, F.R.C.S., assistant surgeon; S. Phillips, M.D., assistant physician; A. Q. Silcock, F.R.C.S., assistant surgeon; lecturers to the nursing staff, and examiners.
M. A. Medill, matron.

**St. George's Hospital.**

Hugh M. Macpherson, F.R.C.S., chairman of the Committee of Nursing; Charles T. Dent, F.R.C.S., assistant surgeon, lecturer to the nurses.

**St. Marylebone Infirmary and Training School.**

John R. Lunn, F.R.C.S., medical superintendent.
Elizabeth Vincent, matron.

Other institutions have this memorial under consideration, and names are being sent in to Dr. Steele, Guy's Hospital, S.E., from whom any information may be obtained on application.

*(To be continued.)*

